# Low-Reflectivity Drusen With Overlying RPE Damage Revealed by Spectral-Domain OCT: Hint for the Development of Age-Related Macular Degeneration

**DOI:** 10.3389/fmed.2021.706502

**Published:** 2021-10-26

**Authors:** Shasha Yang, Zongyin Gao, Haijiang Qiu, Chengguo Zuo, Lan Mi, Hui Xiao, Xing Liu

**Affiliations:** ^1^Department of Ophthalmology, Guangzhou First People's Hospital, School of Medicine, South China University of Technology, Guangzhou, China; ^2^State Key Laboratory of Ophthalmology, Zhongshan Ophthalmic Center, Sun Yat-sen University, Guangzhou, China

**Keywords:** drusen, age-related macular degeneration (AMD), optical coherence tomography, character, predictor

## Abstract

**Purpose:** To observe the relationship between the characteristic changes in the drusen morphology revealed by the spectral-domain optical coherence tomography (SD-OCT) and the progression of age-related macular degeneration (AMD).

**Methods:** A total of 380 drusen in 45 eyes in 35 patients with the intermediate drusen were longitudinally followed up every 6 months by SD-OCT for a period of 24 months. The drusen were divided into the dynamic group and stable group according to the following parameters: number, volume, concurrent retinal pigment epithelium (RPE)/ellipsoid zone (EZ) damage, and the development of advanced AMD. The morphological characteristics of the progressive or stable drusen were further analyzed. Odds ratios (ORs) and the risk for the drusen progression were calculated.

**Results:** The level of interobserver and intraobserver agreement for each drusen tomographic morphological parameters ranged from 82.7 to 90%. At the end of an average follow-up of 15.92 ± 6.99 months, six patients developed choroidal neovascularization and no patients developed geographic atrophy. Finally, 139 drusen changed and 241 drusen remained stable. The drusen with low reflectivity (*p* < 0.001; OR: 5.26; 95% CI: 2.24–12.36), non-homogeneity without a core (*p* < 0.001; OR: 4.31; 95% CI: 2.08–8.92), RPE damage (*p* < 0.001; OR: 28.12; 95% CI: 9.43–83.85), and the EZ damage (*p* < 0.001; OR: 14.01; 95% CI: 5.28–37.18) were significantly associated with active change; the drusen with low reflectivity (*p* = 0.01; OR: 2.95; 95% CI: 1.29–6.75) and decreased overlying RPE reflectivity (*p* < 0.001; OR: 21.67; 95% CI: 9.20–51.02) were the independent predictors for progression. The drusen with high reflectivity were significantly associated with stabilization (*p* = 0.03; OR: 0.17; 95% CI: 0.04–0.84).

**Conclusion:** Spectral-domain optical coherence tomography is an optimized, accurate, and efficient method to follow-up the drusen. The intermediate non-exudative AMD prognosis of the patient was most strongly correlated with the drusen reflectivity and disruption of the overlying RPE layer. The drusen with low reflectivity and overlying RPE damage were more likely to progress and required frequent follow-up.

## Introduction

Age-related macular degeneration (AMD) is the leading cause of vision loss among people over 60 years of age in the developed countries (1). With vision loss, the life of a person with AMD becomes inconvenient and elderly people with AMD have a higher risk of depression and anxiety than unaffected elderly people (2). Because there are no vision-restoring therapies, the individuals with intermediate AMD are just as anxious about losing vision as the individuals who are actually blind (3). Thus, it is crucial to find the predictors of intermediate AMD progression, which is only slightly understood at present.

Macular drusen are the characteristic lesions of intermediate AMD (4). Therefore, the size and number of the drusen are used for AMD staging and for predicting the likelihood of the disease progression and vision loss. Color fundus photographs are commonly used to evaluate the morphology of the drusen over time (5–10). However, the color images do not provide detailed information about the changing internal morphology of the drusen (11). There is usually a long process before the drusen progress to advanced AMD. Historically, based on the color photographs, the large drusen and pigmentary changes have been identified as the risk factors for progression (7). However, these factors do not provide enough information to allow a clear understanding of the course of the disease to be achieved. The introduction of spectral-domain optical coherence tomography (SD-OCT) has enabled the investigation of the ultrastructural morphological characteristics of the drusen *in vivo* and the distinction of subclasses of the drusen types (12–15). SD-OCT can provide important information, which will facilitate further understanding of the disease pathogenesis, identification of the potential risk factors for progression to advanced AMD (16), and the development of early interventions before disease progression to the advanced stages.

## Methods

### Patients

We enrolled 35 patients with binocular AMD and eventually excluded eyes with choroidal neovascularization (CNV) or geographic atrophy (GA) at baseline. The mean age of the patients was 69.94 ± 7.30 years, ranging from 55 to 81 years. We defined early AMD as the presence of at least one drusen on any of the B-scan (6 × 6 mm) centered on the fovea on OCT (12). This study was approved by the Human Ethics Committee of Zhongshan Ophthalmic Center and informed consent was obtained from all the participants in accordance with the Declaration of Helsinki.

The study participants had to be able to fix their eyes for at least 1 min to allow adequate SD-OCT scanning. At the beginning of the follow-up period, the eligibility criteria were as follows: (1) age between 50 and 85 years; (2) bilateral large drusen (125 μm) or large drusen in one eye and advanced AMD in the fellow eye (17); (3) the drusen within 3,000 μm of the foveola; and (4) no signs of any other retinopathy or advanced AMD in the study eye. The exclusion criteria were as follows: (1) bilateral late-stage AMD, including a large area (>1,500 μm) of central GA or CNV; (2) significant opacity of the cornea or media; (3) amblyopia; (4) retinal abnormalities other than AMD; (5); diabetes; (6) uncontrolled hypertension (systolic blood pressure > 150 mm Hg and diastolic blood pressure > 90 mm Hg); (7) neurological or systemic disease that could compromise vision; (8) use of medication known to affect the retina (e.g., hydroxychloroquine); (9) use of antioxidant agent one month prior to study entry and during the period of follow-up; and (10) physical and/or mental impairment or inability to sign a consent form. None of the participants had a history of high myopia, ocular trauma, or ocular surgery.

### Image Acquisition

All the study participants were examined five times during a six month interval within a follow-up period of 24 months. At each visit, visual acuity was recorded with the Early Treatment Diabetic Retinopathy Study (ETDRS) charts. SD-OCT images were obtained with a Spectralis™ OCT system (Heidelberg Engineering, Germany) after pupil dilation with 0.5% tropicamide and 5% phenylephrine. A standardized SD-OCT scan (λ = 870 nm; 40,000 A-scans/s; 38 B-scans) was performed in each study eye with a transverse resolution of 6 μm and an axial resolution of 5 μm. Because of the independent pairs of the scanning mirrors, the eye movements were automatically corrected by the in-system “eye tracking” device, which enabled the identification of the same scanning location during the follow-up visits. To increase the image quality, the automatic real-time analysis scanning of the same location was performed and the images were averaged to reduce the signal-to-noise ratio. The automatic real-time averaging settings were 7 and the scan quality was 23–36 dB (15, 18).

### Spectral Domain-Optical CT Classification of the Drusen Ultrastructure

The images were always evaluated as white-on-black in the HEYEX software. In high-speed mode, the vertical presentation of the OCT scan was magnified twice (19). The graded and analyzed morphological characteristics of the drusen included shape, internal reflectivity, homogeneity, and status of the retinal pigment epithelium (RPE) or ellipsoid zone (EZ) layer above the drusen (20–23). The shape of the drusen was classified as domed, saw-toothed, pointed, or cuticular. The internal reflectivity of the drusen was graded as a low, medium, or high compared with the reflectivity of the adjacent RPE layer on the same scan. Homogeneity was evaluated and classified as follows: non-homogeneous without a core (a distinct focus of hyperreflectivity inside the drusen), non-homogeneous with a central core, or homogeneous (relatively uniform internal reflectivity). The status of the RPE layer and EZ layer above the drusen was determined to be intact/present or disrupted/absent.

The SD-OCT characteristics of the drusen were analyzed by two trained independent graders (SSY and LM) and a senior grader (XL) decided in the case of a discrepancy. Interobserver reliability was determined for each parameter by calculating Cohen's kappa coefficient.

### Image Analysis

The SD-OCT characteristics of the drusen were graded and analyzed every six months. The drusen were divided into the dynamic group and the stable group according to the follow-up results.

(1) Dynamic group: An increase or decrease in the number or volume of the drusen over time, the appearance or expansion of RPE damage, the appearance or expansion of the EZ damage scope, or progression into CNV or GA.

(2) Stable group: After 24 months of follow-up, no obvious changes in the number or volume of the drusen, no increase in the RPE or EZ damage, and no development of CNV or GA.

### Statistical Analysis

Statistical analysis was performed by using SPSS Statistical Software version 20 (Chicago, Illinois, USA). The prevalence of each basic morphological pattern was calculated and analyzed with descriptive statistics. The chi-squared test was used for the analysis of the categorical data. Enumeration data were analyzed by *t*-test. The strength of the association of the different drusen characteristics between the dynamic drusen group and the stable drusen group is shown as the odds ratio (OR) with 95% CI. For all the tests, a *p* < 0.05 was considered statistically significant.

## Results

A total of 380 drusen were observed in 45 eyes of 35 patients. The mean age of the patients was 69.94 ± 7.30 years, ranging from 55 to 81 years; 8 patients were women and 27 patients were men. The mean follow-up period was 15.92 ± 6.99 months (range: 3–27 months). At the end of the follow-up period, 139 drusen had changed and 241 drusen had remained stable. During the follow-up period, the number of the drusen increased in 13 eyes and decreased in 2 eyes; the volume of the drusen increased in 18 eyes and decreased in 3 eyes; the range of RPE damage increased in 21 eyes; and the range of the EZ damage increased in 20 eyes. There were no cases of a decrease in the range of the RPE or EZ damage. In addition, there were six cases (six eyes) of CNV development and no cases of GA development. Table 1 shows the shape, internal reflectivity, homogeneity, RPE status, and EZ integrity in the dynamic and stable drusen groups.

**Table 1:** Summary of the drusen basic morphological parameters seen in the drusen dynamic group and the drusen stable group.

		**Dynamic drusen**		**Stable drusen**	
		**No**.	**%**	**No**.	**%**
**Shape**					
Dome	244	112	45.9	132	54.1
Saw-toothed	66	14	21.2	52	78.8
Pointed	47	13	27.7	34	72.3
Cuticular	23	0	0	23	100
**Reflectivity**					
Low	91	70	76.9	21	23.1
Medium	202	65	32.2	137	67.8
High	87	4	4.6	83	95.4
**Homogeneity**					
Homogeneous	290	71	24.5	219	75.5
Nonhomogeneous with a core	7	3	42.9	4	57.1
Nonhomogeneous without a core	83	65	78.3	18	21.7
**RPE damage**					
Present	129	114	88.4	15	11.6
Absent	251	25	10.0	226	90.0
**EZ damage**					
Present	77	74	96.1	3	3.9
Absent	303	65	21.5	238	78.5

*RPE, retinal pigment epithelium; EZ, ellipsoid zone*.

**Figure 1:**
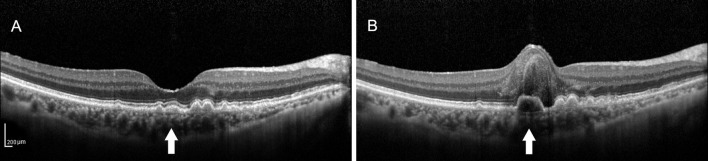
**(A)** Spectral-domain optical coherence tomography (SD-OCT) image of a 64-year-old female dry patient with age-related macular degeneration (AMD) showing the medium-reflectivity drusen without the retinal pigment epithelium (RPE) or ellipsoid zone (EZ) damage at the fovea. **(B)** SD-OCT image of the same patient 1.5 years later, demonstrating the notable changes in the fovea, including retinal neuroepithelial hemorrhagic detachment with the local RPE and EZ disruption, which were associated with choroidal neovascularization (CNV). Note that the detected drusen (white arrow) developed into the low-reflectivity fusion drusen corresponding to the presence of the above changes.

**Figure 2:**
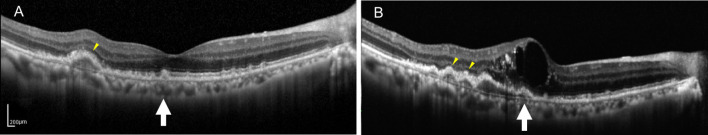
**(A)** SD-OCT image of a 75-year-old male dry AMD patient showing the multiple, medium-reflectivity drusen with the extensive RPE and EZ damage surrounding the fovea. **(B)** SD-OCT image of the same patient 1 year later, demonstrating retinal neuroepithelial serous detachment with the additional and expanded drusen in his right macula. Note that the detected drusen (yellow arrowheads and white arrow) developed into the low-reflectivity confluent drusen corresponding to the presence of the above changes. Note that some drusen (white arrow) showed low reflectivity under the hemorrhagic shadow, whereas others appearing hyporeflective (yellow arrowheads) that were not covered by the hemorrhagic shadow.

At the end of the follow-up period, the drusen in approximately 60% (21/35) of eyes and 62.2% (28/45) of eyes had changed. Among them, there were six cases (six eyes) of neovascular AMD development. All the eyes had the drusen with RPE damage and five eyes had the drusen with the EZ damage. In the six development eyes of neovascular AMD, we noticed an interesting phenomenon: there were no changes in the drusen shape or homogeneity on SD-OCT, but in three cases, the medium-reflectivity drusen converted to the low-reflectivity drusen (Figures 1, 2). These may be due to the internal reflectivity change of the drusen or due to the greater thickness and shadowing of the hemorrhagic change on the appearance of the underlying drusen.

Univariate analysis showed that the low-reflectivity drusen (OR: 5.26, *p* < 0.001), the non-homogeneous drusen without a core (OR: 4.31, *p* < 0.001), the drusen with RPE damage (OR: 28.12, *p* < 0.001), and the drusen with the EZ damage (OR: 14.01, *p* < 0.001) were associated with progression. Furthermore, multivariate analysis [generalized estimating equation (GEE) model] showed that the low-reflectivity drusen and the drusen with RPE damage were independent predictors for progression with OR values of 2.95 and 21.67, while the high-reflectivity drusen were less likely to progress with an OR value of only 0.17 (Table 2).

**Table 2:** The generalized estimating equation (GEE) model analysis of the drusen progression factors.

**Influencing factors**	**Univariate analysis**		**Multivariate analysis**	
	**OR (95% CI)**	***P***	**OR (95% CI)**	***P***
Age (per year)	1.06 (1.00–1.12)	0.066	–	–
Gender (Male vs. Female)	1.99 (0.84–4.74)	0.120	–	–
Reflectivity (Low vs. Medium)	5.26 (2.24–12.36)	<0.001	2.95 (1.29–6.75)	0.010
Reflectivity (High vs. Medium)	0.48 (0.21–1.07)	0.074	0.17 (0.04–0.84)	0.030
Homogeneity (Nonhomogeneous with a core vs. Homogeneous)	2.52 (0.48–13.23)	0.275	–	–
Homogeneity (Nonhomogeneous without a core vs. Homogeneous)	4.31 (2.08–8.92)	<0.001	–	–
RPE damage (Present vs. Absent)	28.12 (9.43–83.85)	<0.001	21.67 (9.20–51.02)	<0.001
EZ damage (Present vs. Absent)	14.01 (5.28–37.18)	<0.001	2.72 (0.91–8.14)	0.074

*RPE, retinal pigment epithelium; EZ, ellipsoid zone*.

The interobserver and intraobserver agreement of the drusen grading data is shown in Table 3. In summary, the interobserver agreement was highest for reflectivity (95.2%) followed by shape (93.8%), presence of RPE damage (91.5%), presence of the EZ damage (89.7%), and homogeneity (83.4%). The intraobserver agreement was similar in the two groups. Observer 1 had a slightly higher intraobserver agreement compared to observer 2.

**Table 3:** Interobserver and intraobserver agreement in the drusen grading of the morphological parameters.

**Agreement**	**Shape**		**Reflectivity**		**Homogeneity**		**RPE damage**		**EZ damage**	
Interobserver	93.8		95.2		83.4		91.5		89.7	
	Obs1	Obs2	Obs1	Obs2	Obs1	Obs2	Obs1	Obs2	Obs1	Obs2
Intraobserver	94.1	92.0	94.8	91.6	88.9	82.7	94.4	91.2	90.1	87.3

*Obs1, observer 1; Obs2, observer 2*.

## Discussion

Drusen are an important quantifier of AMD severity. Drusen contain a great diversity of the compounds that appear to influence the complex pathogenesis of AMD (24, 25). Different clinical subtypes of the drusen have been described in AMD. Large (≥125 μm, width of a large vein at the disk margin) drusen with a soft, fuzzy boundary and pigmentary abnormalities are the well-recognized predictors for the progression to advanced AMD (26). However, these predictors are mainly based on the color fundus photography findings. However, the changes taking place within these high-risk drusen are unknown.

**Figure 3:**
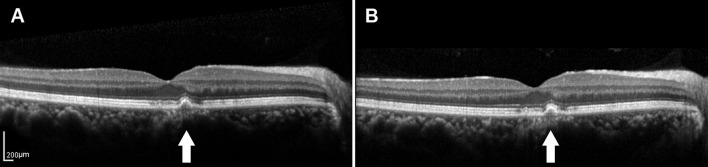
**(A)** SD-OCT image of a 65-year-old male dry AMD patient showing the medium-reflectivity drusen without the RPE damage and the EZ is focally thinned at the fovea. **(B)** SD-OCT image of the same patient 1.5 years later, demonstrating the changes in neither the number nor the volume of the drusen. White arrows showed the drusen remained stable during the follow-up.

**Figure 4:**
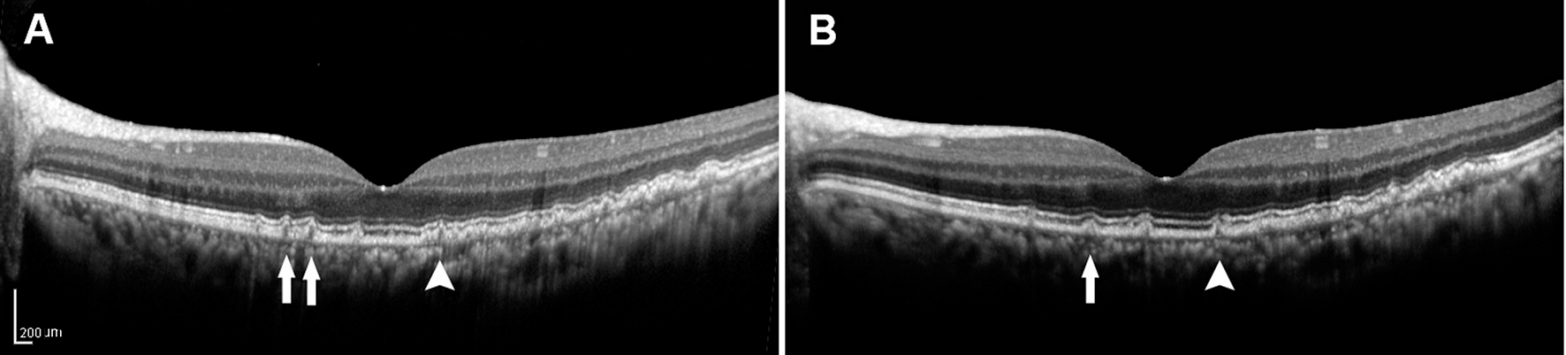
**(A)** SD-OCT image of a 57-year-old female dry AMD patient showing the cuticular drusen without the RPE or the EZ damage around the fovea. **(B)** SD-OCT image of the same patient 2 years later, demonstrating a decrease in the number (arrow) and volume (arrowhead) of the drusen. SD-OCT, spectral domain-optical coherence tomography; AMD, age-related macular degeneration; RPE, retinal pigment epithelium; EZ, ellipsoid zone.

Recent developments in retinal imaging, especially with the introduction of high-resolution SD-OCT, have expanded the understanding of the complex ultrastructure of the drusen. Based on the SD-OCT morphological characteristics, 17 distinct subclasses of the drusen types were described by Khanifar et al. (20, 21). Approximately, 50% of the soft-indistinct drusen on color photography have a different appearance on SD-OCT (16, 27–29). We propose that investigating changes in the appearance of the various characteristics within the drusen *via* SD-OCT may contribute to a better understanding of the genotype, severity of disease, and risk of progression in AMD. They are consistent with that SD-OCT can provide more information about the risk factors for progression based on color photography described by Khanifar et al. (21). In this study, we used longitudinal SD-OCT photography to investigate the dynamic process of the drusen development to provide a clinically useful judgment regarding the risk of progression to vision-threatening AMD in the patients with the drusen. Drusen with low reflectivity and an overlying RPE or the EZ layer with decreased reflectivity were more likely to progress, whereas the high-reflectivity drusen were more likely to remain stable (Figures 3, 4).

There was considerable interobserver and intraobserver agreement in the grading of the drusen according to the morphological parameters on SD-OCT compared with the grading of the drusen according to the findings on color photography. In the Wisconsin age-related maculopathy grading system, the interobserver agreement for the type of the drusen was 70.6% and the intraobserver agreement for the type of the drusen was 62.5% (30). The Age-Related Eye Disease Study (AREDS) research group found that the reproducibility of the type of the drusen ranges from 59.0 to 77.5% (exact agreement) (31). In this study, there were only two readers and they were from the same center and the reading standards were unified before reading; thus, our interobserver agreement was different from that reported by the Classification of Atrophy Meetings (CAM) group (32) (by 12 readers from 6 reading centers). In this study, the level of interobserver and intraobserver agreement for each morphological parameter was generally more than 90% for the drusen reflectivity, shape, and RPE damage, while the drusen homogeneity and the EZ damage showed the lowest agreement at 82.7%.

In this study, out of the 380 drusen, approximately one-third (139/380) were the dynamic drusen and two-thirds (241/380) were the stable drusen. Univariate analysis regarding the differences in the SD-OCT morphological characteristics between the dynamic and stable drusen showed that the low-reflectivity drusen, the non-homogeneous drusen without a core, the drusen with RPE damage, and the drusen with the EZ damage were more likely to be the dynamic drusen (*p* < 0.001).

Further multivariate analysis showed that the low-reflectivity drusen and the drusen with RPE damage were the independent predictors for AMD progression. In particular, the progressive OR of the low-reflectivity drusen was 2.95, while the high-reflectivity drusen was only 0.17 indicating that the low-reflectivity drusen were most likely to progress into advanced AMD and the high-reflectivity drusen were most likely to remain stable. These findings are consistent that the transition to a low internal reflectivity in the drusen is a dangerous signal of a transition toward CNV.

We found that all the six patients with the drusen with RPE damage eventually developed neovascular AMD including five patients with the drusen with the EZ damage. Multivariate analysis also showed that the progressive OR of the drusen with RPE damage over the drusen without RPE damage was 21.67. This is consistent with the traditional concept that RPE impairment significantly contributes to AMD (33–35). Ellipsoid corresponds to the outer part of the cone photoreceptor of the inner segments that are packed with the mitochondria and shows a continuous, high-reflection signal layer located over the RPE layer on SD-OCT (34, 36). The integrity of the EZ is an important indicator of the structural integrity of the photoreceptor. Disruption of the EZ caused by the various fundus diseases has been proven to be obviously correlated with abnormal photoreceptor function (37–39). Since the apical side of the RPE cells is in intimate contact with the outer segments of the photoreceptor and the drusen with RPE damage were independent predictors for AMD progression, drusen with the overlying EZ disruption ought to be a reasonable predictor for AMD progression. However, the *p*-value of this factor slightly exceeded 0.05 in the GEE analysis. However, the result could be different if more eyes were included in this study. We did not observe a decrease in the range of the RPE or EZ damage in any case suggesting that the RPE or EZ damage might be irreversible.

Sarks (40) studied the drusen by using panoramic electron microscopy and proved that the clinical drusen phenotypes differed in ultrastructure and, thus, in composition. He found that the amorphous substance in the small hard drusen was dense. When the small hard drusen grew larger than approximately 63 μm in size, the amorphous content became less compact. When the drusen reached confluence, the content of the drusen broke down and the particles of the amorphous material were of a smaller size. Histologically, the confluent soft drusen contain granular material apparently derived from the breakdown of the small hard drusen. When the globules break down further to very finely granular material, the drusen appeared empty or very finely granular on electron microscopy. On the other hand, Sarks (40) also noted that as the content breakdown, the drusen tended to lose both the sharp boundary and the nodular surface elevation, whereas the RPE over the broken-down drusen seemed to disappear, the photoreceptors appeared stunted, and there was a loss of overlying photoreceptor nuclei. Curcio proposed that the soft drusen/basal linear deposits (BLinDs) and subretinal drusenoid deposits (SDDs) confer the risk for end-stage atrophy and neovascularization. The life cycle of the soft drusen includes growth, anterior migration of RPE atop drusen, collapse, and atrophy (41, 42).

Lipid particles accumulate between the RPE and Bruch's membrane and form the drusen (43, 44). Due to the age-related changes, apoB100 lipoprotein accumulates in Bruch's membrane. Furthermore, the RPE synthesizes and secretes very-low-density lipoprotein (VLDL) and low-density lipoprotein (LDL) (45). Failure of the choriocapillaris–Bruch's membrane complex to clear the normal RPE secretions to plasma can lead to the accumulation of the drusen (40, 41, 45, 46). Differences in the lipid particle composition are most likely to result in the differences in internal reflectivity and the changes in the lipid particle composition within the drusen may be a sign of a poor prognosis for the drusen.

Vande Ven et al. (47) studied 19 eyes of 10 patients with the basal laminar drusen by using SD-OCT and found that the small hard drusen were subjected to the constant changes in volume during four months of follow-up. Small hard drusen with decreased reflectivity of the overlying RPE or photoreceptor layer were more likely to show volume regression, whereas the pointed, small hard drusen were more likely to show volume progression. The term basal laminar drusen has been superseded by the cuticular drusen (23). Balaratnasingam et al. (23) considered that the ultrastructural characteristics of the cuticular drusen appear similar to those of the hard drusen; however, their life cycle and macular complications are more comparable to those of the soft drusen. With more than five years of follow-up, the neovascularization and GA occurred at a frequency of 12.5 and 25% cuticular drusen eyes, respectively. In this study, the shape of the drusen was unrelated to the notable changes in SD-OCT in the subjects, which may be due to the relatively small sample size and a short follow-up period.

The limitations of this study include the consideration of SDDs accumulating in a different layer of the outer retina, appearing differently in the terms of retinal topography, and having a different pathological basis and pathogenicity than the drusen; therefore, the SDDs were not included in this study. As the focus of this article is on the relationship between the internal SD-OCT characteristics of the drusen and the prognosis of AMD, the hyperreflective foci above the drusen were not involved in the analysis. The other limitation of this study is that because the follow-up interval was up to six months, the study missed capturing the moment when the reflectivity of the drusen changed before the transformation into the neovascular lesions. Therefore, these results need to be confirmed in a clinical cohort study with larger sample size and a longer follow-up period with shorter follow-up intervals.

This study demonstrated the differences between the drusen that were stable and those that were dynamic, even with the relatively small sample size. To the best of our knowledge, this is the first prospective cohort study on the drusen in Chinese people.

In conclusion, the findings of this study suggest that the prognosis of the drusen is most strongly correlated with the reflectivity of the drusen and disruption of the overlying RPE layer. For the patients with the drusen of low inner reflectivity and concurrently decreased reflectivity of the overlying RPE and EZ layers on SD-OCT, an increased follow-up frequency is strongly recommended to avoid missing the opportunity of optimum treatment.

## Data Availability Statement

The original contributions presented in the study are included in the article/supplementary material, further inquiries can be directed to the corresponding author/s.

## Ethics Statement

The studies involving human participants were reviewed and approved by the Human Ethics Committee of Zhongshan Ophthalmic Center. The patients/participants provided their written informed consent to participate in this study. Written informed consent was obtained from the individual(s) for the publication of any potentially identifiable images or data included in this article.

## Author Contributions

SY: study design, acquisition of data, analysis and interpretation of data, and drafting of the manuscript. XL: study design, analysis and interpretation of data, drafting of the manuscript, and critical revision. HX: analysis and interpretation of data. LM: acquisition of data and analysis and interpretation of data. HQ: recruiting patients and analysis and interpretation of data. ZG: study design, recruiting patients, and analysis and interpretation of data. CZ: drafting of the manuscript and critical revision. All authors contributed to the article and approved the submitted version.

## Funding

This study was supported by the National Natural Science Foundation of China (Grant Number: 81970808), the Fundamental Research Funds of the State Key Laboratory of Ophthalmology and the Sun Yat-sen University Clinical Research 5010 Program (Grant Number: 2014016), the Guangzhou General Science and Technology Project of Medicine and Health (Grant Number: 20161A011007), and the Science Foundation of Guangzhou First People's Hospital (Grant Number: M2019018).

## Conflict of Interest

The authors declare that the research was conducted in the absence of any commercial or financial relationships that could be construed as a potential conflict of interest.

## Publisher's Note

All claims expressed in this article are solely those of the authors and do not necessarily represent those of their affiliated organizations, or those of the publisher, the editors and the reviewers. Any product that may be evaluated in this article, or claim that may be made by its manufacturer, is not guaranteed or endorsed by the publisher.
